# Glioblastoma: From Pathophysiology to Novel Therapeutic Approaches

**DOI:** 10.3390/biomedicines13081963

**Published:** 2025-08-12

**Authors:** Anatevka Ribeiro, Gianna Fote, Alexander Himstead, Michelle Zheng, Emma Elliott, Sara Mae Smith, Jerry Lou, Carlen A. Yuen

**Affiliations:** 1 Department of Neurology, Division of Neuro-Oncology, University of California, Irvine, CA 92697, USA; 2 Chao Family Comprehensive Cancer Center, University of California, Irvine, CA 92697, USA; 3 Department of Neurological Surgery, University of California, Irvine, CA 92697, USA; 4 UC Irvine Charlie Dunlop School of Biological Sciences, University of California, Irvine, CA 92697, USA; 5 Department of Psychology, University of California, San Diego, La Jolla, CA 92093, USA; 6 Department of Pathology, University of California, Irvine, CA 92697, USA

**Keywords:** glioblastoma, vaccine, immunotherapy, laser interstitial thermal therapy (LITT), GammaTile^®^, chimeric antigen receptor (CAR) T-cell therapy, theranostics

## Abstract

Glioblastoma (GBM) is the most common and aggressive primary malignant brain tumor. Despite the current standard of care therapy, including maximal surgical resection, chemoradiation, and tumor-treating fields, prognosis remains poor. Therapeutic failure is driven by an immunosuppressive tumor microenvironment, poor drug penetration across the blood–brain barrier, and robust resistance mechanisms. Epigenetic alterations further compound treatment resistance by enhancing DNA repair and promoting survival pathways. Molecular profiling has identified key prognostic and predictive biomarkers. Gene expression analyses have delineated GBM subtypes, each with distinct molecular features and therapeutic vulnerabilities that hinder successful clinical translation. This review integrates the pathophysiological, diagnostic, and therapeutic landscape of GBM to inform of future strategies for improved patient outcomes.

## 1. Introduction

Glioblastoma (GBM) is the most common and aggressive primary malignant brain tumor [1]. Prognosis is poor, with a median overall survival (mOS) of 14.6 to 20 months [1]. The current standard of care (SOC) includes maximal safe surgical resection followed by radiotherapy, chemotherapy, and tumor-treating fields (TTFs) [1,2]. Despite this multi-modal approach, GBM is uniformly fatal.

**Figure 1:**
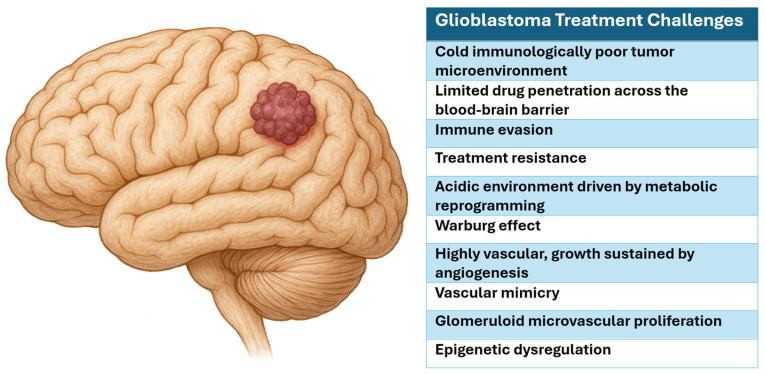
Glioblastoma treatment challenges.

Multiple factors contribute to therapeutic failure, including a “cold” immunologically poor tumor microenvironment (TME), limited drug penetration across the blood–brain barrier (BBB), immune evasion, and treatment resistance (Figure 1) [3]. Epigenetic alterations and heterogeneity exacerbate these processes by promoting aberrant DNA repair, activating pro-survival signaling pathways, and upregulating drug efflux transporters [4]. Prior advances in genomic and epigenomic profiling have led to the identification of prognostic and predictive biomarkers, including isocitrate dehydrogenase (*IDH*) mutations and O^6^-methylguanine-DNA methyltransferase (MGMT) promoter hypermethylation [5,6]. MGMT encodes a DNA repair enzyme that removes alkyl groups from the O6 position of guanine, thereby reversing the cytotoxic lesions induced by temozolomide (TMZ) [7,8,9]. MGMT promoter methylation silences its expression and impairs DNA repair, which subsequently enhances tumor sensitivity to alkylating agents [7,8,9]. MGMT promoter methylation serves as a key predictive and prognostic biomarker for TMZ [7,8,9].

Criteria for diagnosing GBM are increasingly reliant upon molecular alterations. Alongside classic histopathological features of microvascular proliferation and necrosis, the 2021 World Health Organization (WHO) Classification of CNS Tumors introduces an integrated diagnostic approach, which incorporates genetic alterations [10]. Presence of an *IDH* wild-type status and either epidermal growth factor receptor (*EGFR*) amplification, telomere reverse transcriptase (*TERT*) promoter mutations, or +7/−10 chromosomal alterations is sufficient to diagnose a tumor as GBM, even in the absence of classic histological features, including necrosis and microvascular proliferation [10]. Molecular diagnostics techniques, including next-generation sequencing, fluorescence in situ hybridization (FISH), and methylation profiling, are adjunctive tools used to accurately diagnose GBMs [11]. One notable exception is histologically low-grade *IDH*-wild-type gliomas with isolated *TERT* promoter mutation, which have a statistically significantly better prognosis than classic *IDH*-wild-type GBM, arguing for caution when using isolated *TERT* promoter mutation as an independent diagnostic criterion [12].

Gene expression profiling has defined three GBM subtypes (classical, mesenchymal, and proneural) [13]. Each subtype is associated with distinct molecular drivers and therapeutic vulnerabilities [13]. The classical subtype is enriched for *EGFR* amplification and chromosome +7/−10 [13,14]. *EGFR*-targeted therapies are potential treatments, but BBB penetration and intratumoral heterogeneity are barriers to efficacy [13]. The classical subtype and *EGFR* amplification may be informative for therapeutic stratification and have been associated with shorter progression-free survival (PFS) in recurrent (r) GBM treated with bevacizumab (BEV). The mesenchymal subtype frequently harbors *NF1* loss and an inflammatory signature [13,14,15,16]. This subtype has demonstrated increased sensitivity to immune-modulating approaches, including checkpoint inhibitors, though success has been modest [17]. The proneural subtype, often characterized by *PDGFRA* alterations and tumor protein (*TP*)*53* mutations, has demonstrated limited responsiveness to conventional therapies, but may benefit from targeted inhibition of *PI3K/AKT/mTOR* or DNA damage response pathways [13]. Despite these insights, clinical translation remains challenging due to the heterogeneous nature of GBMs, resistance mechanisms, and the lack of reliable biomarkers for therapeutic stratification.

Existing reviews chiefly focus on the pathophysiology, diagnosis, or treatment of GBM in isolation. The significant aspect of this study is the identification of the pathophysiological aspects of GBM to better direct more effective diagnostics and therapeutics for GBM (Table 1).

## 2. Glioblastoma Pathophysiology

### 2.1. Glioma Stem Cells

Glioma stem cells (GSCs) have been implicated as the tumor-initiating population in the origin of GBM [18]. The presence of key genetic alterations in GSCs is also frequently found in GBM, including mutations in *TP53*, *EGFR*, and phosphatase and tensin homolog (*PTEN*), thereby supporting the notion that GSCs give rise to GBM [10,19,20,21]. Moreover, the pronounced heterogeneity of GBM reflects the inherent plasticity of GSCs, including their ability to differentiate, dedifferentiate, and adapt to the diverse TME [22,23,24].

**Figure 2:**
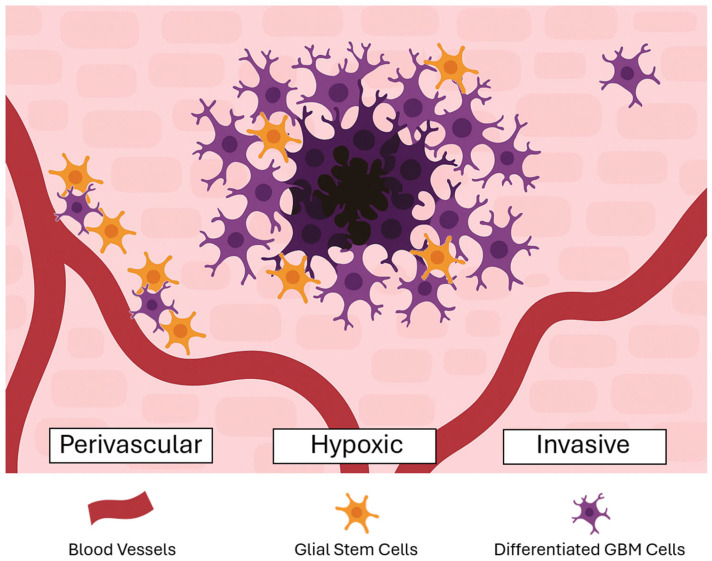
Glioma stem cell perivascular, hypoxic, and invasive niches.

Within the TME, GSCs are sustained by three specialized niches—the hypoxic, invasive, and perivascular niches—with each providing distinct molecular cues that promote GSC maintenance, self-renewal, and tumor propagation (Figure 2) [25,26,27,28]. The hypoxic environment of GBM is primarily localized within the necrotic core, where limited oxygen availability and poor vascularization hinder effective delivery of chemoradiation [28,29]. In response to hypoxia, GBM cells upregulate vascular endothelial growth factor (VEGF), which stimulates angiogenesis and contributes to the formation of abnormal, leaky vasculature. This process supports tumor survival under oxygen-deprived conditions and facilitates tumor invasion and progression by remodeling the surrounding TME [29].

Within the hypoxic niche, hypoxia-inducible factor 1-alpha (HIF-1α) is stabilized and initiates transcriptional programs that support tumor invasion and survival [30,31]. Astrocytes, adjacent to endothelial cells and pericytes, promote tumor invasion using short-range cell-cell signaling [32,33]. Astrocytes also upregulate the expression of zinc finger E-box-binding homeobox 1, a transcription factor implicated in enhancing tumor invasiveness [34,35]. In the perivascular niche, GSCs secrete periostin, a matricellular protein that attracts tumor-associated macrophages (TAMs) [36,37,38]. In turn, TAMs recruit additional macrophages and monocytes, reinforcing the immunosuppressive microenvironment [36,37,38]. Moreover, TAMs produce transforming growth factor-beta, which stimulates the expression of matrix metalloproteinase-9, which is a key enzyme involved in extracellular matrix remodeling and GSC proliferation [39,40,41,42,43].

GSCs contribute to treatment resistance through multiple mechanisms. The acquisition of new mutations within GSCs enhances their ability to evade therapies [44]. GSCs also elicit chemo- and radio-resistance by upregulating DNA repair pathways [4,45,46]. This heightened DNA repair capacity includes mechanisms that counteract alkylating chemotherapies, particularly through the expression of MGMT, whose promoter is typically unmethylated in GSCs [7,47,48,49]. Moreover, GSCs overexpress ATP-binding cassette transporters, which promote drug efflux and reduces intracellular drug concentrations, thereby contributing to chemoresistance [50,51]. GSC-directed treatment strategies are directed at inhibiting the metabolic and developmental pathways, including the *Notch* and Hedgehog pathways, and enhancing immune response [52,53].

### 2.2. Tumor Microenvironment

**Figure 3:**
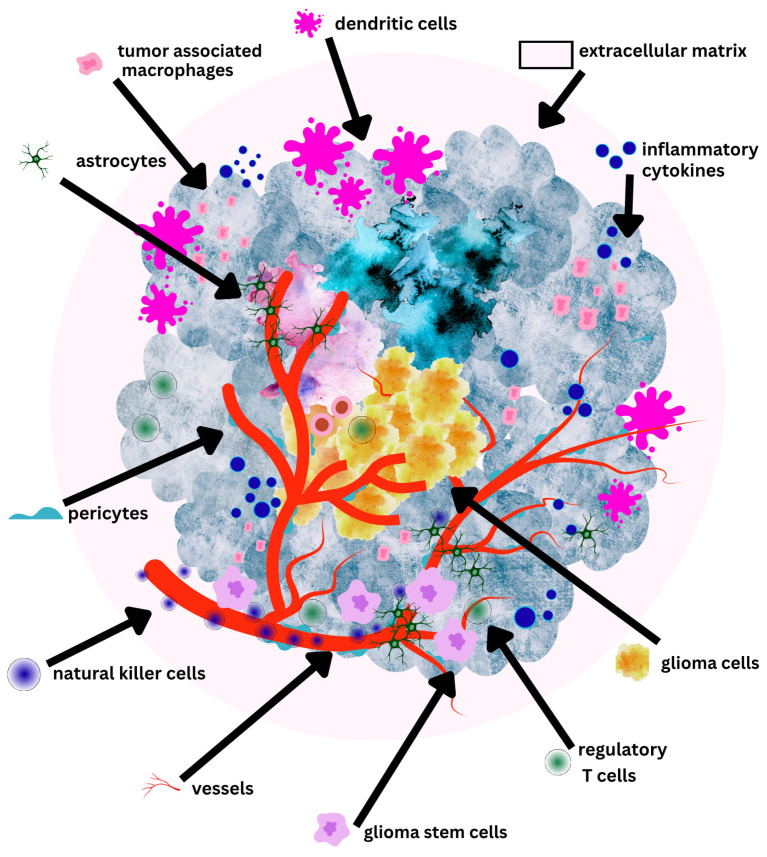
Glioblastoma tumor microenvironment components.

The GBM TME is immunologically “cold” and profoundly immunosuppressive (Figure 3) [54,55]. While the central nervous system (CNS) was historically considered to be an immune-privileged site due to the restrictive nature of the BBB, the discovery of the CNS lymphatic drainage challenged this widely accepted notion [56,57].

GBMs actively modify the TME to promote tumor survival and immune evasion [58,59]. One key mechanism involves the polarization of macrophages toward an M2 phenotype, which suppresses T cell activation and promotes T cell exhaustion [60,61]. Through the release of cytokines and chemokines, GBMs reprogram macrophages from a pro-inflammatory, tumor-fighting M1 phenotype to an anti-inflammatory, tumor-supportive M2 state [36,62]. Glioma-associated macrophages further inhibit T cell function and upregulate immune checkpoints, including programmed cell death-ligand 1 (PD-L1) [63].

The TME is also acidic and devoid of oxygen [29,64]. Both hypoxic and acidic conditions induce HIF1α, impairing mitotic arrest and enhancing DNA damage repair pathways that decrease GBM susceptibility to ionizing radiation [65]. This acidic environment results from increased glycolysis and lactate production, driven by metabolic reprogramming [53]. Overexpression of proton and lactate transporters, such as monocarboxylate transporters, drives the export of hydrogen ions and lactate into the extracellular space, thereby lowering the extracellular pH [66,67]. The harsh acidic pH conditions contribute to GSC maintenance by promoting the stemness phenotype in glioma cells through the expression of CD133+ and HIF2α, which enhances self-renewal and tumorigenesis [27,68]. Moreover, the extracellular acidic pH causes ion trapping and neutralization of weak-base chemotherapies, which reduces the efficacy of these treatments [55,69,70]. The acidic TME contributes to radiation resistance by decreasing radiation-induced DNA damage, inhibiting apoptosis, and driving the invasiveness of these tumors [55,69,71,72]. 

Hypoxia also contributes to the GBM stemness phenotype [73]. In response to hypoxia, GBM cells shift their energy metabolism from oxidative phosphorylation to glycolysis, a phenomenon known as the Warburg effect [74,75]. This metabolic reprogramming permits the continued production of ATP despite limited oxygen [74]. The Warburg effect is reinforced by the upregulation of glucose transporters, including glucose transporter 1, and key glycolytic enzymes, including pyruvate dehydrogenase kinase 1, lactate dehydrogenase A, and hexokinase 2 [53,74]. Additionally, lipid metabolic reprogramming supports the metabolic demands of the hypoxic TME [76,77]. Lipids are a source of energy that GBM channels into pro-oncogenic pathways [74,78]. Lastly, GBMs alter metabolism to increase nucleotide production and resist oxidative stress [79,80]. These metabolic adaptations create the permissive environment under which GBM sustains rapid cell proliferation and survival [81,82]. Inhibition of the glycolytic pathway by targeting glucose transporter 1, pyruvate kinase, and pyruvate dehydrogenase kinase, and hexokinase 2 may shift metabolism from glycolysis to oxidative phosphorylation [53,83].

### 2.3. Angiogenesis

GBMs are highly vascular tumors that rely upon angiogenesis to sustain rapid growth [28,30]. Angiogenesis is the formation of new capillaries from pre-existing blood vessels and is a tightly regulated physiological process [30]. Aberrant neovascularization supplies the essential nutrients, oxygen, and metabolic substrates needed for GBMs to sustain growth [84]. Angiogenesis in GBM is orchestrated by multiple factors. First, hypoxia triggers the stabilization and activation of HIF-1α and the subsequent expression of VEGF [85,86]. Excessive production of VEGF and pro-angiogenic factors, including angiopoietins, platelet-derived growth factor, and fibroblast growth factors, results in abnormal vascular permeability, the formation of disorganized, dysfunctional vasculature, and BBB disruption [87,88,89]. This BBB disruption contributes to the infiltrative nature and cerebral edema that are characteristic of these tumors [90]. Beyond these angiogenic pathways, GBMs also exploit alternative mechanisms, including vascular mimicry and glomeruloid microvascular proliferation for sustenance [24,91,92]. Vascular mimicry is an endothelial cell-independent process that leads to the formation of vessel-like channels by tumor cells [91]. Early evidence suggests that vascular mimicry is associated with an aggressive glioma phenotype and the vascular heterogeneity of GBMs [91].

### 2.4. Epigenetics

Genetic mutations play a pivotal role in the pathogenesis of GBM. However, emerging evidence underscores the crucial role of epigenetic dysregulation in tumorigenesis, heterogeneity, and therapeutic resistance [93]. Although the precise mechanisms underlying GBM development remain unclear, epigenetic regulators have become promising targets to reverse tumorigenic processes through the restoration of normal gene expression patterns [94,95,96]. Key epigenetic mechanisms include DNA methylation, histone modifications, chromatin remodeling, and microRNA regulation [94,97,98,99,100]. These alterations can lead to the silencing of tumor suppressor genes, activation of oncogenes, and promotion of tumor growth and survival [7].

DNA methylation typically occurs at cytosine–guanine–dinucleotide (CpG) islands near the gene promoter region [6,100]. This process is mediated by DNA methyltransferases, which can suppress gene expression through hypermethylation [101,102]. MGMT promoter methylation has been established as a key prognostic and predictive biomarker for response to TMZ in GBM patients [1,7,103]. However, the optimal cutoff for accurately determining MGMT status remains a matter of debate [8,104].

Histone modifications, including methylation, acetylation, and phosphorylation, regulate chromatin structure and gene accessibility without altering the underlying DNA sequence [105,106]. Dysregulation of histone-modifying enzymes, including histone deacetylases and histone methyltransferases, has been implicated in gliomagenesis [94,107].

Chromatin-remodeling complexes (CRCs) control gene expression, DNA repair, and the cell cycle by altering nucleosome positioning [97,98,99]. Mutations in CRC components can disrupt the nucleosome structure, leading to aberrant gene expression and tumor progression [99].

## 3. Glioblastoma Diagnostic Challenges

Distinguishing viable tumors from treatment-related effects can prove to be challenging on magnetic resonance imaging (MRI). Pseudoprogression, a treatment-related effect, typically occurs within the initial three months following concurrent chemoradiotherapy and has the appearance of enlarging or new contrast-enhancing lesions [108]. Radiation necrosis is a delayed treatment-related effect occurring months to years following chemoradiation and is another phenomenon that contributes to the challenge of diagnosing rGBM [108]. Accurate distinction between these effects and viable tumor is crucial for treatment-making decisions. MRI perfusion, MR spectroscopy, and amino acid positron emission tomography (PET) (Table 2) are adjunctive diagnostic imaging techniques to improve upon diagnostic accuracy. However, despite these advancements, tissue diagnosis remains the gold standard.

### 3.1. 2-Deoxy-2-[^18^F]fluoro-D-glucose

2-deoxy-2-[18F]fluoro-D-glucose ([^18^F]FDG) is widely used for tumor detection in multiple cancers [109]. [^18^F]FDG PET leverages the elevated glycolytic activity that is characteristic of tumors to differentiate neoplastic from non-neoplastic tissue [109]. However, the high physiological uptake of [^18^F]FDG in normal gray matter significantly limits the tumor-to-background contrast in the brain, thereby complicating the delineation of tumor from normal brain [109]. Furthermore, [^18^F]FDG lacks specificity in distinguishing GBM from inflammatory or infectious processes, thereby restricting its routine use for GBM [110,111]. 

### 3.2. ^18^F-Floretyrosine (FET PET)

^18^F-floretyrosine (TLX101-CDx, Pixclara^®^) is an amino acid PET tracer targeting large amino acid transporter 1/2 that is overexpressed in glioma cells [112]. Preliminary evidence for the accuracy of FET PET in identifying glioma cells is promising [113,114,115]. The sensitivity and specificity of the tumor-to-brain ratio max (ranging from 1.9 to 2.3) are 91% and 84%, respectively [116]. However, increased uptake in the setting of seizures limits the diagnostic accuracy [110]. In a small series of nine tissue-confirmed rGBM patients, Ceccon et al. assert that all FET PET images obtained for these patients showed increased uptake [114]. In addition to its diagnostic role, FET PET may have the potential to play a prognostic role in identifying GBM patients with a short-term survival of 12 months or less [117].

The FIG trial (ACTRN12619001735145) is currently underway to determine the utility of ^18^F-floretyrosine in differentiating viable tumor from pseudoprogression and to assess a potential role in radiation planning [118].

### 3.3. [^68^Ga]Ga-PSMA-617

[^68^Ga]Ga-prostate-specific membrane antigen (PSMA)-617 radioligand was originally developed for targeting PSMA in prostate cancer. Preliminary evidence for [^68^Ga]Ga-PSMA-617 has garnered interest in its use for identifying GBM [119,120]. [^68^Ga]Ga-PSMA-617 shows promise in detecting areas of neoangiogenesis within regions of low metabolic activity [119,120]. Detection of tumor angiogenesis rather than tumor cells directly can potentially enable high-contrast lesion detection in regions of disrupted BBB, including in the neovasculature of GBM [119,121]. The advantage of this radioligand lies in the low background uptake in normal brain tissue, which enhances the conspicuity compared to traditional agents, including [^18^F]FDG [119,120]. However, the application of [^68^Ga]Ga-PSMA-617 in GBM remains investigational [119,120]. Limitations for use in GBM include heterogeneous PSMA expression amongst GBMs, and poor uptake in tumors with minimal neovascularization [119,120]. PSMA-targeting radioligands may accumulate in areas of neoangiogenesis, thereby leading to poor specificity and false positives in cases of metastatic disease or inflammation [119,120]. Lastly, existing evidence is limited to small cohort studies and case series.

## 4. Newly Diagnosed Glioblastoma Therapies and Challenges

### 4.1. Current Therapies

#### 4.1.1. Surgery

Surgical resection is indicated as the first step of a multimodal treatment plan in the majority of GBM cases in order to obtain a tissue diagnosis and achieve cytoreduction. For patients who are not candidates for open surgery or have tumors located in eloquent brain regions, frame-based or frameless stereotactic biopsy serves as a reliable alternative, offering high diagnostic accuracy with minimal morbidity [122,123]. The goal of surgical management in GBM is to achieve maximal safe resection to remove as much of the mass as possible without inducing a permanent neurological deficit [124]. Numerous studies have shown a positive correlation between the extent of resection (EOR) and OS, with at least 95% resection necessary to confer a survival benefit [125,126]. However, residual tumor volume (RTV) of less than 2 cm^3^ has emerged as a potentially more robust predictor of outcome [125,127]. Some studies do not corroborate these findings, and RTV as a stronger prognostic factor over EOR remains a subject of debate [126]. Additionally, some studies suggest that preoperative tumor volume is associated with survival outcomes [125]. Although subsequent studies have not consistently replicated these findings, the principle of maximal safe resection remains the cornerstone of GBM surgery [128,129,130].

Patients who develop surgery-related neurological deficits are less likely to receive adjuvant chemoradiation, further underscoring the importance of preserving neurological function. Deficits occur primarily as a result of attempted resection in areas of the tumor in eloquent structures or from perilesional infarcts from the sacrifice of blood vessels. Resection of extensive lesions throughout the dominant hemisphere may produce a language deficit. Similarly, resection of lesions that cross into both hemispheres (“butterfly gliomas”) through either the corpus callosum or commissures is associated with a risk for motor deficits (4.3%), abulia (2.5%), and a survival benefit of only 6 months [123,124].

To enable maximal safe resection, a range of advanced intraoperative technologies has been integrated into surgical practice. Image-guided navigation systems utilize preoperative MRI to delineate tumor margins and assist in the precise localization of surgical instruments. Intraoperative imaging modalities, including MRI and ultrasound, offer real-time visualization during resection, enhancing surgical accuracy [131]. However, intraoperative MRI (IoMRI) is associated with high costs, limited availability, and increased operative time. In contrast, intraoperative ultrasound (IoUS), while more accessible, demands specialized expertise for accurate interpretation and effective use [131].

Additional tools to support maximal safe resection include preoperative functional MRI and diffusion tensor imaging, which help map eloquent cortical and subcortical regions to avoid critical structures during surgery. Awake craniotomy with intraoperative cortical stimulation further enhances functional preservation by enabling real-time monitoring of patient responses. Another valuable technique is the use of 5-aminolevulinic acid (5-ALA), a fluorescent dye preferentially metabolized by malignant glioma cells. Under ultraviolet light, 5-ALA enables more precise visualization of tumor tissue, facilitating EOR. Its use has been associated with improved surgical outcomes and potential survival benefits while minimizing the risk of postoperative neurological deficits [132].

#### 4.1.2. Stupp Protocol

The SOC for newly diagnosed (nd)GBM is based on the Stupp protocol, which combines TMZ with radiotherapy, followed by adjuvant TMZ. This treatment regimen was established by the pivotal EORTC-NCIC 26981-22981/CE.3 trial (NCT00006353), which evaluated the survival benefit of adding chemotherapy to radiotherapy in GBM patients [1]. Participants were randomized to receive either radiotherapy alone (60 Gy over six weeks) or radiotherapy with concurrent daily TMZ (75 mg/m^2^), followed by 6 cycles of adjuvant TMZ (150–200 mg/m^2^ for 5 days every 28 days) [1]. The addition of TMZ extended mOS from 12.1 months in the radiotherapy-alone group to 14.6 months in the combination therapy group [1]. Moreover, the addition of TMZ increased the two-year survival rate from 10.4% to 26.5%, demonstrating a significant survival advantage and firmly establishing TMZ as a mainstay in the treatment for ndGBM [1].

The optimal duration of adjuvant TMZ in the standard treatment of GBM remains an area of active investigation. The Stupp protocol consists of six cycles of adjuvant TMZ following concurrent chemoradiotherapy [1]. To assess whether extended treatment offers additional benefit, the Phase II GEINO 14-01 randomized trial compared 6 cycles of TMZ to an extended regimen of up to 12 cycles in patients with non-progressive disease [133]. The study found no significant differences in PFS (55.7% vs. 61.3%) or OS between the two groups [133]. However, prolonged TMZ treatment was associated with increased toxicity, including higher rates of lymphopenia and gastrointestinal adverse effects [133].

A schedule of dose-dense (DD) TMZ was investigated in the RTOG 0525 trial, which was designed to overcome MGMT-mediated resistance [134]. In this Phase III study, patients were randomized to receive either standard TMZ or DD TMZ (administered 21 days on, 7 days off) for 6 to 12 cycles [134]. Results showed no significant differences in mOS (16.6 months vs. 14.9 months) or mPFS (5.5 months vs. 6.7 months) [134]. However, results showed greater toxicity with DD TMZ, including higher rates of leukopenia and fatigue [134].

#### 4.1.3. Tumor Treating Fields

Optune™ tumor treating fields (TTF) is an adjunct therapy to chemoradiation for GBM. TTF exerts antitumor effects by disrupting mitosis, promoting apoptosis, and increasing tumor sensitivity to chemotherapy. Transducer arrays placed on the patient’s scalp deliver low-intensity, alternating electric fields for a minimum of 18 hours daily, targeting rapidly dividing GBM cells [2]. The pivotal randomized clinical trial (NCT00916409) demonstrated a significant improvement in both mPFS (6.7 vs. 4.0 months) and mOS (20.9 vs. 16.0 months) with the addition of TTF to adjuvant TMZ [2]. The most common adverse events included scalp irritation and dermatitis [2]. Despite its benefits and favorable safety profile, the trial faced limitations, including the exclusion of patients with poorer prognoses and the lack of a placebo-controlled design using a sham device [2]. The ongoing TRIDENT (EF-32, NCT04471844) trial aims to determine the optimal timing for TTF by evaluating TTF use during chemoradiation and with adjuvant TMZ.

### 4.2. Abandoned Therapies

#### 4.2.1. Carmustine Wafers

Carmustine (BCNU), a nitrosourea-class alkylating agent, inhibits DNA replication and transcription. BCNU formulated into biodegradable polymer wafers (Gliadel^®^ wafers) for direct drug delivery into the tumor resection cavity is FDA-approved for the treatment of GBM [135]. In a Phase III randomized trial, patients with ndGBM who received BCNU wafers at the time of surgery, followed by standard radiotherapy, experienced modest improvement in mOS (13.9 months vs. 11.6 months in those who received placebo) [136]. Despite this survival benefit, long-term follow-up revealed a higher incidence of treatment-related complications, including cerebrospinal fluid (CSF) leaks, elevated intracranial pressure, and seizures [136]. Due to the modest efficacy and risk of adverse events, clinical use of BCNU wafers declined in favor of more effective and better-tolerated therapeutic strategies [137].

#### 4.2.2. Anti-Angiogenic Therapy

BEV is a monoclonal antibody (MAB) that targets VEGF, thereby inhibiting angiogenesis. The placebo-controlled Phase III AVAGLIO trial evaluated the addition of BEV to SOC treatment in ndGBM patients compared to SOC with placebo [138]. The study demonstrated a significant improvement in PFS and quality of life (QoL) with the addition of BEV [138]. However, there was no survival advantage associated with the addition of BEV [138]. Toxicities associated with BEV include hypertension, deep vein thrombosis, pulmonary embolism, and impaired wound healing [138].

Similarly, the Phase III RTOG 0825 trial evaluated the efficacy of BEV in ndGBM [139]. Results from this trial substantiated the lack of OS benefit observed in the AVAGLIO trial [138,139]. The trial also failed to demonstrate a significant improvement in PFS [139]. Although BEV was associated with reduced QoL and neurocognitive decline, it remains unclear whether these cognitive effects were directly attributable to BEV-related neurotoxicity or secondary to disease progression [139]. Consequently, BEV is not recommended as first-line therapy for ndGBM.

The randomized TEMAVIR trial assessed the safety and efficacy of adding neoadjuvant and adjuvant BEV and irinotecan, a topoisomerase I inhibitor, to SOC therapy in patients with nd unresectable GBM [140]. The addition of BEV and irinotecan did not improve OS compared to SOC alone. Moreover, this combination was associated with higher toxicity and increased rates of serious adverse events, including intracranial hemorrhage, gastrointestinal perforation, and thrombotic complications [140]. Given these findings, the combination of BEV and irinotecan with SOC is not recommended for the treatment of unresectable GBM.

## 5. Recurrent Glioblastoma Therapies and Challenges

Despite aggressive treatment with surgery and chemoradiation, local tumor recurrence occurs in approximately 90% of GBM cases within two years. Effective therapeutic options in the recurrent setting remain limited [141].

### 5.1. Current Therapies

#### 5.1.1. Re-Resection

Re-resection may improve OS in patients with gross total re-resected tumors, but re-resection is not broadly indicated for rGBM [142,143]. Select patients with good performance status and an accessible tumor location may benefit from re-resection [144].

#### 5.1.2. Reirradiation

Reirradiation (reRT) is a treatment option for rGBM. While reRT may be safe and effective in certain patients, the criteria for optimal patient selection remain unclear. Favorable factors may include age, higher performance status, planning treatment volume, and longer time interval from prior radiation [145,146].

Advancements in radiation delivery techniques aim to minimize exposure to surrounding healthy brain tissue. However, concerns persist regarding treatment-related toxicities, including cognitive decline and radiation necrosis, particularly in patients with large tumor burden [146]. Reported mOS following SRS or SRT ranges from 6 to 12 months [141]. Some studies suggest that the addition of TMZ or BEV to reRT may offer additional survival benefit. However, it is unclear whether this is due to synergistic effects or patient selection bias [147,148]. The comparative prognostic value of reRT alone versus combined reRT and chemotherapy remains unresolved and is the subject of ongoing investigations [148,149].

#### 5.1.3. Chemotherapy

Lomustine (CCNU) is an alkylating agent that may also inhibit enzymes through amino acid carbamoylation, though its clinical significance remains unclear [150]. The Phase II BELOB (bevacizumab and lomustine in recurrent glioblastoma) trial was designed to assess the safety and efficacy of BEV, CCNU, or combination BEV and CCNU in first recurrence GBM [151]. Following the results from this trial demonstrating a survival advantage with the combination therapy, the Phase III EORTC 26101 trial was initiated following the promising results of the BELOB trial to further assess the combination of BEV and CCNU compared to CCNU alone in these patients [151,152]. While the combination therapy significantly improved PFS with an mPFS of 4.2 months compared to 1.5 months, no significant benefit in OS was noted [152]. Moreover, patients receiving the combination experienced higher rates of treatment-related toxicities compared to those treated with CCNU alone [152].

#### 5.1.4. Anti-Angiogenic Therapy

The Phase II BEV-CPT-11 trial investigated whether or not the cytotoxic properties of irinotecan (CPT-11), a topoisomerase I inhibitor, combined with the anti-angiogenic effects of BEV, could improve outcomes in rGBM compared to BEV alone [153]. Although the combination therapy improved PFS, there was a marginal benefit in OS and notable treatment-related toxicities [153]. As such, single-agent BEV was subsequently FDA-approved for rGBM.

#### 5.1.5. Tumor Treating Fields

The Phase III randomized EF-11 trial compared TTF with the physician’s best choice of chemotherapy in patients with rGBM [2]. The study found no significant difference in mOS between the TTF group and the control group (6.6 months vs. 6.0 months) [154]. However, patients receiving TTF reported better QoL with fewer side effects [2]. A Phase II trial (NCT04221503) is currently underway to evaluate whether combining TTF with niraparib, a PARP inhibitor, can produce synergistic effects and improve outcomes in rGBM.

### 5.2. Abandoned Therapies

The Phase III REGAL trial (REcentin™ in Glioblastoma Alone and with Lomustine) investigated the VEGF inhibitor cediranib as monotherapy and in combination with CCNU compared to CCNU alone in rGBM patients [155]. The study found no significant improvement in PFS or OS across the treatment arms [155]. Furthermore, the cediranib arms were associated with higher rates of toxicity, which resulted in treatment discontinuation for a subset of patients [155]. Given the lack of clinical benefit and an unfavorable safety profile, cediranib is not recommended as monotherapy or in combination with CCNU for rGBM [155].

The Phase II REGOMA trial evaluated regorafenib, a multi-kinase inhibitor, compared with CCNU in rGBM patients [156]. Regorafenib conferred a significant survival advantage with a mOS of 7.4 months compared to 5.6 months with single-agent CCNU [156]. However, regorafenib was associated with higher rates of grade 3 and 4 toxicities, including hypertension and hand-foot syndrome [156]. The subsequent REGOMA-OSS observational study further assessed regorafenib’s efficacy and safety [157]. Findings from this study corroborated the survival benefit observed in the REGOMA trial, but reported a lower incidence of high-grade toxicities [157]. To date, there remains a lack of confirmatory Phase III trials for the efficacy of regorafenib, in addition to concerns regarding toxicity, cost, and limited availability [157]. As such, regorafenib remains investigational for rGBM.

## 6. Novel Therapeutics

### 6.1. Lomustine

The Phase III CeTEG (NOA-09) multicenter, randomized trial evaluated the efficacy of adding CCNU to the SOC in patients with MGMT promoter methylated ndGBM [158]. The addition of CCNU significantly improved mOS compared to SOC alone (48.1 vs. 31.4 months) [158]. While hematologic toxicity was more frequent in the combination group, the regimen was generally well tolerated [158]. Notably, no corresponding improvement in PFS was noted, suggestive of a delayed therapeutic effect [158]. The addition of CCNU to TMZ is currently under investigation (NCT05095376).

### 6.2. Laser Interstitial Thermal Therapy

Laser interstitial thermal therapy (LITT) is a minimally invasive alternative to conventional surgery that uses a laser catheter to deliver targeted thermal ablation to tumor tissue [159,160]. This technique is particularly beneficial for deep-seated or surgically inaccessible gliomas, offering precise tumor destruction while minimizing damage to surrounding healthy brain tissue [160]. LITT may also enhance the effects of chemoradiation, providing a potential synergistic therapeutic benefit [161]. Additional advantages include shorter recovery times, reduced hospital stays, and the ability to initiate adjuvant therapy sooner [161,162,163]. Early studies suggest that LITT is feasible for both nd and rGBM, with potential improvements in QoL [160,164]. Common adverse events include neurological deficits, cerebral edema, hemorrhage, and seizures [160,164]. Results from the LAANTERN study (NCT02392078) reported a median post-procedure survival of 8.97 months in rGBM patients [160]. Future investigations focused on combining LITT with other treatment modalities, including hypofractionated RT and immunotherapy are underway (Table 3).

### 6.3. GammaTile^®^

Brachytherapy offers the advantage of delivering immediate localized radiation therapy. While early studies showed promise, its widespread adoption was hindered by high rates of treatment-related toxicities, including radiation necrosis, wound dehiscence, seed migration, and infections requiring surgical intervention [165,166,167]. Recent innovations have revitalized interest in this modality with a novel design comprised of Cs-131 radiation-emitting seeds embedded within a resorbable collagen-based tile (Table 2) [168]. In 2018, GammaTile^®^ received FDA approval for treating both nd and recurrent malignant intracranial tumors [168,169]. Compared to traditional brachytherapy, GammaTile^®^ demonstrates a more favorable toxicity profile and has emerged as a promising therapeutic for both ndGBM and rGBM patients [170]. In the multi-histology basket trial (NCT03088579), which included 28 rGBM patients, GammaTile^®^ therapy yielded an mOS of 25 months and a low incidence of symptomatic radiation necrosis (7%) [171]. The Phase 4 multicenter Surgically Targeted Radiation Therapy (STaRT) observational trial is currently enrolling ndGBM patients treated with GammaTile^®^ brachytherapy followed by the Stupp protocol (NCT04427384). The ongoing GESTALT trial (NCT05342883) is evaluating GammaTile^®^ as a bridging therapy to initiate treatment between surgical resection and the start of radiation and TMZ, aiming to address early tumor recurrence during this critical time period (Table 4).

### 6.4. Immunotherapy

Programmed cell death protein 1 (PD-1) is an immune checkpoint receptor expressed on T cells to maintain self-tolerance by limiting the immune response [172]. GBMs exploit this pathway by expressing the PD-L1 ligand to induce immunosuppression and evade immune surveillance [173]. MABs targeting PD-1 (i.e., pembrolizumab and nivolumab) and PD-L1 (i.e., atezolizumab and avelumab) are collectively referred to as immune checkpoint inhibitors (ICIs). Although ICIs have transformed the treatment landscape for cancers such as melanoma and non-small cell lung cancer, their success in GBM has been limited [174,175,176,177,178].

The CheckMate trials, including CheckMate 143, 498, and 548, evaluated the efficacy of nivolumab for the treatment of GBM [176,177,178]. The CheckMate 498 and 548 trials assessed nivolumab for ndGBM in combination with radiotherapy with or without TMZ, but failed to demonstrate any survival benefit with the addition of ICIs [177,178]. Similarly, the Phase III CheckMate 143 trial compared nivolumab to BEV in rGBM patients, but results were equally disappointing [176]. These studies highlight the unique immunosuppressive TME, low mutational burden, and limited immune cell infiltration, by which substantial barriers exist to promote immune activation in GBMs [179]. Furthermore, ICIs can trigger nonspecific immune activation, leading to immune-related adverse events, which may affect multiple organ systems, including the skin, gastrointestinal tract, lungs, pituitary gland, and, rarely, the CNS [180,181].

### 6.5. Targeted Radionuclide Therapy

Targeted radionuclide therapy is a precision-based theranostic approach offering both diagnostic and localized therapy to target and bind to tumor cells, while sparing the normal surrounding brain.

One such target is the L-type amino acid transporter (LAT1), which is highly expressed in GBM cells [182]. LAT1 facilitates the transport of phenylalanine and drives the uptake of O-(2-[^18^F]fluoroethyl)-L-tyrosine [(^18^F)FET] in GBM [112]. As a diagnostic tool, [^18^F]FET) PET identifies LAT1 overexpression for diagnosis [112]. As a therapeutic, [131I]IPA (iodine-131 4-iodo-l-phenylalanine) is cytotoxic to targeted and neighboring cells [183]. A Phase I trial (NCT05450744) is currently evaluating the safety and efficacy of ^131^I-TLX101 in combination with SOC therapy for ndGBM. Additionally, a separate Phase I trial (NCT03849105) assessing 4-l-[^131^I]iodo-phenylalanine alongside radiation therapy in rGBM reported a mOS of 13 months, with the treatment deemed as safe and well tolerated [183].

Other forms of TRTs include peptide receptor radionuclide therapy (PRRT), nanoparticles, and antibody-based radionuclide therapy targeting tumor-specific antigens, including *EGFR* and *EGFRvIII*. PRRTs, including somatostatin analogs, have been successful for the treatment of neuroendocrine tumors due to the ubiquitous expression of somatostatin receptor 2 (SSTR2) [184,185]. In stark contrast to neuroendocrine tumors, SSTR2 is not highly expressed in GBM [186,187], which may limit the success of PRRT with somatostatin analogs. Moreover, antibody-based radionuclide therapy faces challenges with tumor heterogeneity and limited BBB penetration. However, nanoparticles may overcome these BBB limitations [188,189,190]. Preclinical evidence suggests that gold nanoparticles act as a radiosensitizer, immune activator, and generator of heat and radical oxygen species [190,191].

### 6.6. Vaccines

Tumor-directed vaccines represent another promising form of personalized therapy for GBM (Table 5) and are categorized as either cell-based or antigen-based approaches [192]. However, the identification of tumor-specific antigens remains challenging due to the heterogeneity of GBM [193]. The most common adverse events are pruritus and reactions at the injection site, though Grade 3 allergic reactions and anaphylaxis can occur [194].

#### 6.6.1. Dendritic Cell Vaccines

Dendritic cells (DCs) are antigen-presenting cells (APCs) that stimulate robust adaptive immune responses [3]. This capability spurred the development of a DC-based vaccine in the Phase III NCT00045968 trial that assessed DCVax^®^-L in combination with SOC therapy in nd or rGBM patients compared to matched external controls who received SOC alone [195]. The trial demonstrated a mOS of 19.3 months in the DCVax^®^-L group compared to 16.5 months in the control group, with corresponding 5-year survival rates of 13.0% and 5.7%, respectively [195]. However, the use of an external control group for comparison and the absence of *IDH* mutation status in a subset of patients may have confounded these results [196]. Additionally, these results have not been consistently replicated in other studies, including the ICT-107 Phase IIb trial evaluating the efficacy of a DC vaccine pulsed with 6 glioma-associated peptides that are commonly expressed in GBM (human epidermal growth factor (*HER*)2, interleukin (IL)13Rα2, TRP-2, gp100, MAGE-1, and AIM-2) in ndGBM patients. No significant difference in OS was found between ICT-107 vs. autologous DCs without peptide-loading. However, an exploratory subgroup analysis was suggestive of a survival benefit in HLA-A2–positive patients [197]. Challenges to the success of DC vaccines for the treatment of GBM have been attributed to the immunosuppressive TME and suboptimal antigen loading [198,199]. Current strategies are focused on optimizing loading DCs with personalized neoantigens, combinatorial therapy with ICIs, and combining DC vaccines with immune checkpoint inhibitors [198].

#### 6.6.2. Peptide Vaccines

Peptides derived from tumor-specific mutations serve as the foundation for peptide vaccines [200]. These synthetic peptides mimic tumor antigens to stimulate immune responses [200]. APCs present these peptides to T cells that circulate to the tumor to exert cytotoxic effects [200].

SurVaxM is a peptide vaccine that targets survivin, an anti-apoptotic protein abundantly expressed in GBM, but is largely absent in normal brain [201]. Results from the Phase II NCT02455557 trial demonstrated that the addition of SurVaxM to TMZ led to an increased mPFS of 11.4 months and a mOS of 25.9 months, compared to historical controls [201]. Notably, patients who developed high anti-SurVaxM antibody titers experienced longer OS, independent of MGMT promoter methylation status [201]. Building on these encouraging results, a randomized, placebo-controlled Phase II SURVIVE trial (NCT05163080) is currently underway to evaluate whether the addition of SurVaxM to TMZ improves outcomes in ndGBM patients.

An *EGFRvIII* variant is present in approximately 30% of GBMs [202,203]. Rindopepimut (CDX-110) was designed to target this variant in ndGBM patients [204]. The early-phase ReAct trial showed encouraging results, with the speculation that the loss of *EGFRvIII* expression was due to successful immune-mediated tumor eradication [204]. However, these findings were not confirmed in the Phase III ACT IV trial, which failed to demonstrate a survival benefit [205]. As a result, the study was terminated, highlighting the challenges of interpreting early immunotherapeutic success.

### 6.7. CAR T-Cell Therapy

Chimeric antigen receptor (CAR) T-cell therapy involves re-engineering the patient’s own T-cells to recognize and eradicate tumor cells [206]. While this approach has shown remarkable success in hematologic cancers, its efficacy in solid tumors (including GBM) remains limited [207,208]. Challenges include GBM’s highly immunosuppressive TME, tumoral heterogeneity, and anatomical barriers [209,210,211]. To overcome these hurdles, bivalent CAR T-cells, locoregional delivery strategies, and combination therapies with ICIs are being explored [212,213,214]. Targets including IL13Rα2, EGFRvIII, and HER2 are under investigation for the treatment of GBMs (Table 6) [209,215,216].

#### 6.7.1. IL-13Rα2

IL-13 is a cytokine involved in regulating immune responses. Its receptor subunit, IL-13Rα2, has emerged as a promising target due to its selective overexpression in GBM and minimal expression in normal tissues [203,217]. In a landmark case study, repetitive intracavitary and intraventricular IL13Rα2 CAR T cell administration in a GBM patient resulted in significant regression of intracranial and spinal lesions, with no off-tumor toxicities. The response was not durable due to the heterogeneity of antigens, T cell exhaustion, and immunosuppression [209]. A Phase I trial investigating IL-13Rα2-targeted CAR-T cells in 41 rGBM using three different locoregional routes of administration, including intratumoral, intraventricular, and dual intratumoral/intraventricular, found that IL-13Rα2-CAR-T cell therapy was safe and feasible with no dose-limiting toxicities. A subset of patients experienced improved QoL and prolonged stable disease [217]. Those patients who received dual intratumoral/intraventricular delivery showed significant OS, suggesting that dual-route locoregional delivery may optimize CAR-T trafficking and activity, although further mechanistic studies are needed [217].

#### 6.7.2. *EGFR*

Preliminary results from the Phase I NCT05168423 trial, which was designed with a bivalent CAR T-cell therapy targeting both *EGFR* and IL-13Rα2 in rGBM, showed that 75% of the six enrolled patients maintained stable disease at two months post-treatment [211]. Furthermore, fifty percent of these patients experienced a measurable reduction in tumor volume, highlighting the potential of a multi-targeted CAR T-cell approach in GBM therapy.

*EGFRvIII* CAR T-cells have been investigated in rGBM (NCT02209376). Results from this trial showed that CAR T cells trafficked to the tumor, but reduced *EGFRvIII* expression, inhibitory signals, and regulatory T cell infiltration were observed in the TME [218]. Accordingly, while CAR T cells can migrate to the GBM site, therapeutic resistance may be limited by antigen loss and immune resistance [218]. These findings were confirmed in the Phase 1 NCT03726515 trial, wherein combined *EGFRvIII* CAR T-cells and pembrolizumab showed notable changes in the TME, including T cell exhaustion and presence of regulatory T cells, which resulted in an mPFS of 5.2 months and a mOS of 11.8 months [213].

#### 6.7.3. *HER2*

In the Phase 1 NCT05168423 trial, patients with recurrent *HER2*-positive GBM treated with *HER2*-CAR virus-specific T cells showed an mOS of 11.1 months from infusion and 24.5 months from initial diagnosis [219]. In a Phase I study, intracranial injection of *HER2*-targeted CAR-NK cells in nine patients with rGBM was found to be safe and feasible. In this study, 5 patients achieved stable disease [220]. Tandem CAR T-cell therapy offers a promising strategy to address the limitations of single-antigen targeting by enhancing efficacy and reducing antigen escape. Dual CAR T cells targeting both *HER2* and IL13Rα2 have demonstrated improved tumor control and delayed progression compared to single-target CAR T-cell approaches [221].

#### 6.7.4. CAR Natural Killer-Cell Therapy

NK cells are essential components of both the innate and adaptive immunity. Unlike T cells, NK cells do not require antigen priming to exert potent anti-tumor cytotoxic effects [222]. CAR NK-cell therapy is an emerging approach to GBM treatment with engineered NK cells expressing CAR T-cells targeting *HER2* (NCT03383978) and *EGFRvIII* [218,222,223]. Compared to CAR T-cell therapies, CAR NK-cell approaches offer the advantages of a lower risk of cytokine release syndrome and the potential for standardized, off-the-shelf manufacturing [224,225,226]. Nevertheless, key challenges persist. In addition to the immunosuppressive TME, limited persistence in vivo and the complex, labor-intensive manufacturing process are additional barriers for CAR NK-cell therapy to reach maximum potential [222].

## 7. Future Directions

### 7.1. Diagnostics

GBM diagnostic strategies are shifting toward precise molecular subtyping and the identification of actionable targets for personalized therapeutic decision-making. Radiogenomic models are positioned to stratify and potentially guide surgical planning [227]. Advanced machine learning algorithms applied to multiparametric MRI can non-invasively infer key molecular alterations such as *IDH* mutation, *MGMT* promoter methylation, and *EGFR* amplification, enabling preoperative risk assessment and tailored surgical strategies aimed at maximizing resection while minimizing morbidity [227,228].

In parallel, liquid biopsy approaches utilizing tumor-derived cell-free DNA (cfDNA) from CSF offer a minimally invasive avenue for monitoring tumor dynamics [229,230]. CSF-derived cfDNA has demonstrated superior sensitivity compared to plasma in detecting glioma-specific alterations, including *IDH* and *TP53*. CSF-derived cfDNA findings may inform of real-time tumor burden and may be an early indicator of progression or resistance to existing treatment [229,230].

These innovative diagnostics represent a shift toward precision-guided management of GBM, with the potential to improve monitoring of these tumors and early detection of recurrent disease.

### 7.2. Gene Therapy

Gene therapy aims to alter disease progression by introducing or modifying genetic material by employing strategies of suicide gene delivery, oncolytic viral therapy, immunomodulatory gene therapy, and CRISPR-Cas9 gene editing [231]. Viral vectors (lentiviruses, adenoviruses, herpes simplex virus (HSV), and retroviruses), direct therapeutic gene delivery into GBM cells.

Suicide gene therapy using retroviral vectors was among the earliest clinical approaches for gene therapy. In a Phase III trial conducted by Rainov et al., HSV-thymidine kinase via a retrovirus, followed by ganciclovir treatment, was deemed safe, but did not improve OS, likely due to suboptimal gene delivery [232]. Similarly, the Phase III Toca 511 study that was designed to assess a retroviral vector consisting of cytosine deaminase that converted 5-fluorocytosine (5-FU) to 5-fluorouracil (5-FC), failed to show a survival benefit [233].

Oncolytic adenoviruses and related viral vectors are engineered to selectively infect and replicate in tumor cells, triggering direct oncolysis and enhancing anti-tumor immunity by releasing tumor-associated antigens. Several oncolytic viruses are under active investigation in GBM. Pelareorep is a reovirus-based therapy that replicates in cells with activated Ras signaling, promoting both direct cytotoxicity and systemic immune activation [234]. TG6002 is a vaccine virus that is engineered to fuse with tumor cell membranes and convert 5-FU into 5-FC [235]. H-1 parvovirus (H-1PV) is a single-stranded DNA virus that enters GBM cells via clathrin-mediated endocytosis, causing DNA damage [236]. In the Phase I/IIa ParvOryx01 trial, H-1PV penetrated the BBB, infiltrated tumor tissue, and was found to be safe with signals of immune activation [236]. The PVS-RIPO recombinant oncolytic poliovirus targets the CD155 poliovirus receptor that is overexpressed in GBM [237]. Results from the Phase II trial investigating its use demonstrated extended survival in a subset of patients [237].

CRISPR-Cas9 gene editing has emerged as a novel tool to improve our understanding of GBM biology and has exposed new therapeutic targets. Genomics screening utilizing CRISPR-Cas9 gene editing can uncover oncogenic drivers, including those involved in epigenetic regulation, angiogenesis, immune evasion, and DNA damage repair [238,239]. CRISPR-Cas9 screens in *EGFRvIII*-expressing GBM cells have unveiled the transcription factor E2F6 as a key driver of TMZ resistance and may represent a viable therapeutic target [240]. *ATRX* is frequently mutated in GBM, and CRISPR-Cas9 *ATRX* knockout suppresses cell proliferation, invasion, and vasculogenic mimicry in glioma cells, resulting in increased sensitivity to TMZ by enhancing TMZ-induced DNA damage and apoptosis [241]. Consequently, *ATRX* is a potential prognostic marker and therapeutic target for GBM [241]. CRISPR-Cas9 has uncovered genes involved in angiogenesis. CRISPR-Cas9 knockdown of *Notch1* in U251 and U87MG cells impaired angiogenesis [242]. Preliminary evidence shows that CRISPR-Cas9 PDPN knockout, a cell surface protein thought to be involved in lymphangiogenesis, is responsive to immune checkpoint blockade [243,244]. Within the realm of DNA damage repair, CRISPR-Cas9 downregulation of MGMT expression has been found to reverse TMZ resistance in glioblastoma cell lines [245]. CRISPR-Cas9 gene editing is a promising therapeutic for GBM. However, vehicle delivery, off-target effects, and immunogenicity are challenges that need to be addressed prior to clinical application [246].

### 7.3. Light-Based Therapy

#### 7.3.1. Photodynamic Therapy

Photodynamic therapy (PDT) is a promising adjunctive modality for the management of GBM [247]. PDT induces cytotoxicity by generating reactive oxygen species (ROS) through intracranial delivery of light and selective photosensitizer accumulation in tumor tissue with subsequent activation by specific wavelengths of light [247,248,249]. Using fiber optics, spatially confined activation minimizes damage to the surrounding eloquent cortex [247]. Photosensitizers, including 5-ALA, provide several advantages, including preferential uptake by glioma cells and conversion to protoporphyrin IX, providing both intraoperative fluorescence guidance and tumor-selective cytotoxic potential when exposed to light [248,250]. Moreover, PDT disrupts tumor vasculature and induces immunogenic cell death, which may enhance antitumor immunity [248]. Early-phase trials have shown improved local control and PFS, particularly in patients with residual tumor volume [248,251]. Limitations include heterogeneous photosensitizer uptake and light attenuation in deep tissue [248,249,251].

#### 7.3.2. Sonodynamic Therapy

In contrast to PDT, sonodynamic therapy (SDT) leverages the deep penetrance of ultrasound to activate the sensitizer even in surgically inaccessible tumor regions, with reduced off-target effects [252,253]. Like PDT, SDT is a non-invasive treatment modality that combines the tumor-selective accumulation of a sonosensitizer, including 5-ALA, with focused ultrasound to produce selective cytotoxic ROS in tumor tissue [253]. Preclinical data demonstrate pronounced tumor cell apoptosis, BBB modulation, and vascular disruption [252]. A phase 0 first-in-human trial (NCT04559685) assessed the safety and biological response in recurrent high-grade gliomas through escalating-dose MRgFUS and SONALA-001 (5-ALA). In this trial, the feasibility of the treatment with histologic evidence of therapy-induced cell death without significant adverse events was confirmed [254].

## 8. Conclusions

Glioblastoma is a highly aggressive primary tumor of the central nervous system, characterized by a poor prognosis despite standard treatments, including surgery, radiation, and chemotherapy. Emerging therapeutic approaches, including immunotherapies, anticancer vaccines, GammaTile, laser interstitial thermal therapy, and targeted radionuclide therapy, offer promising avenues to overcome the resistance mechanisms that have historically limited treatment efficacy. These innovative strategies may hold the potential to significantly improve patient outcomes. However, further validation through well-designed clinical trials is essential to establish their therapeutic value [192].

## Figures and Tables

**Table 1:** Glioblastoma pathophysiologic features and corresponding therapeutic strategies.

Resistance Mechanism	Description	Therapeutic Strategy
BBB	Limits penetration of therapeutic agents	Focused ultrasound BBB penetrable drug Nanoparticle-based delivery
Glioma stem cells	Subpopulation of self-renewing therapeutic-resistant cells	*Notch*, *Wnt*, or Hedgehog pathway inhibitors Anti-CD133 or CD44 therapies
The Warburg Effect	Glycolysis for ATP production	Glucose transporter 1 inhibitors Pyruvate kinase inhibitors Pyruvate dehydrogenase kinase inhibitors Hexokinase 2 inhibitors
Immune evasion	Immunosuppressive tumor microenvironment	Immune checkpoint inhibitors CAR-T cells Vaccines
Angiogenesis	Aberrant blood vessel formation	Anti-VEGF therapy
Epigenetics	MGMT promoter methylation	Alkylating therapy
Genomic alterations	Alterations that promote resistance, proliferation, and survival	Targeted therapy CRISPR-based gene editing

**Table 2:** Glioblastoma and radiotracer trials (recruiting as of 25 July 2025).

Clinical Trial Identification	Study Title	Diagnostic Test
NCT06451042	FET-PET/MRI Based Treatment Planning for Glioblastoma Multiforme in Post-Surgical Patients (FET-TREAT)	FET-PET/MRI
NCT02902757	FDG PET/CT in Monitoring Very Early Therapy Response in Patients with Glioblastoma	fludeoxyglucose F-18
NCT06613841	Multitracer [^18^F]Fluciclovine and 18F-FDG PET, and Advanced MRI for Metabolic Profiling of Glioblastoma	fluciclovine F18
NCT07067905	Clinical Evaluation of [^68^Ga]Ga-XT771 PET for Diagnosis in Patients with Glioblastoma and Clear Cell Renal Cell Carcinoma	68Ga-XT771
NCT06645808	PET-imaging of Two Vartumabs in Patients with Solid Tumors	
NCT06797661	Insights Into the Pathophysiology of Neurovascular Uncoupling in Patients with Brain Lesions.	FDG-PET
NCT06113705	Imaging and Biological Markers for Prediction and Identification of Glioblastoma Pseudoprogression: a Prospective Study.	18F-GE-180 PET
NCT06319027	Identifying Findings on Brain Scans That Could Help Make Better Predictions About Brain Cancer Progression, The GABLE Trial	fluciclovine F18
NCT06645808	PET-imaging of Two Vartumabs in Patients with Solid Tumors	89Zr-DFO-N-Suc-F8scFv 89Zr-DFO-N-Suc-C9scFv
NCT05781321	Short Course Radiotherapy for the Treatment of Patients With Glioblastoma, SAGA Study	fluorodopa F 18
NCT05386043	Registering Genomics and Imaging of Tumors (ReGIT)	FET F-18

**Table 3:** Glioblastoma and LITT trials (recruiting as of 25 June 2025).

Clinical Trial Identification	Primary Objective	Phase	Main Inclusion Criteria	Newly Diagnosed (N) or Recurrent (R)
NCT04699773	Evaluate the effects of LITT combined with hypo-fractionated radiation therapy on newly diagnosed gliomas	NA	Glioma|Glioblastoma|Brain Tumor	N
NCT04181684	Evaluate the efficacy of LITT combined with hypo-fractionated radiation therapy in treating recurrent gliomas	NA	Glioblastoma|Brain Tumor|Glioma|Neoplasms	R
NCT03277638	Assess the safety and efficacy of combining LITT with pembrolizumab in patients with brain tumors	I/II	Glioblastoma, Adult	R
NCT06558214	Evaluate the safety and feasibility of TTFields, MLA, and pembrolizumab combination therapy in recurrent or progressive WHO Grade IV gliomas	II	Recurrent Glioblastoma	R

NA = not applicable.

**Table 4:** Glioblastoma and GammaTile trials (recruiting as of 25 June 2025).

Clinical Trial Identification	Primary Objective	Phase	Main Inclusion Criterion	Newly Diagnosed (N) or Recurrent (R)
NCT05342883	Evaluate the feasibility and safety of adding GammaTile at resection with standard chemoradiation in newly diagnosed glioblastoma	IV	Glioblastoma	N
NCT04427384	Evaluate real-world clinical and patient-reported outcomes to determine the effectiveness and safety of STaRT therapy	NA	Brain Tumor, Recurrent|Brain Tumor|Brain Tumor, Primary|Brain Tumor—Metastatic|Brain Tumor, Adult: Glioblastoma|Brain Tumor, Adult Meningioma	R

NA = not applicable.

**Table 5:** Glioblastoma vaccine trials (recruiting as of 25 June 2025).

Clinical Trial Identification	Primary Objective	Phase	Main Inclusion Criterion	Newly Diagnosed (N) or Recurrent (R)
NCT02287428	Evaluate NeoVax with radiation, pembrolizumab, and temozolomide in newly diagnosed glioblastoma.	I	Glioblastoma	N
NCT06805305	Assess DOC1021 + pIFN with standard care in newly diagnosed glioblastoma.	II	Glioblastoma (glioblastoma)	N
NCT05743595	Test a neoantigen DNA vaccine with PD-1 blockade in MGMT-unmethylated glioblastoma.	I	Unmethylated Glioblastoma	N
NCT04573140	Determine safety and MTD of RNA-LP vaccines in adult glioblastoma and pediatric HGG.	I|II	Adult Glioblastoma|High Grade Glioma|WHO Grade III or IV Malignant Glioma	N
NCT06389591	Evaluate safety and MTD of RNA-LP vaccines in recurrent glioblastoma.	I	Recurrent Glioblastoma	R
NCT04201873	Assess pembrolizumab combined with ATL-DC vaccine in surgically accessible recurrent glioblastoma.	I	Recurrent Glioblastoma	R
NCT03382977	Evaluate safety and tolerability of VBI-1901 in recurrent glioblastoma.	I|II	Glioblastoma	R

**Table 6:** Glioblastoma CAR-T trials (recruiting as of 25 June 2025).

Clinical Trial Identification	Primary Objective	Phase	Main Inclusion Criterion	Newly Diagnosed (N) or Recurrent (R)
NCT06186401	Evaluate the safety, side effects, and optimal dose of E-SYNC CAR T cells following lymphodepleting chemotherapy in EGFRvIII-positive glioblastoma.	I	EGFR Gene Mutation|Glioblastoma|MGMT-Unmethylated Glioblastoma|Recurrent Glioblastoma	R
NCT05660369	Evaluate the safety and efficacy of IL15-enhanced GPC3-CAR T cells (GO-CART) in patients with GPC3-positive brain tumors.	I	Glioblastoma|Malignant Glioma|Recurrent Glioblastoma|Recurrent Glioma	R
NCT04003649	Assess the safety and effectiveness of IL13Rα2-CAR T cells alone or with nivolumab and ipilimumab in recurrent or refractory glioblastoma.	I	Recurrent Glioblastoma|Refractory Glioblastoma	R
NCT06482905	Evaluate the safety, tolerability, and antitumor activity of anti-B7-H3 CAR-T cells (TX103) in recurrent or progressive grade 4 glioma.	I	High-grade Glioma|WHO Grade IV Glioma	R
NCT06186401	Assess the safety, side effects, and optimal dose of EGFRvIII-targeting CAR T cells (E-SYNC) following lymphodepleting chemotherapy in EGFRvIII+ glioblastoma.	I	EGFR Gene Mutation|Glioblastoma|MGMT-Unmethylated Glioblastoma|Recurrent Glioblastoma	R
NCT05835687	Determine the maximum tolerated dose and safety of locoregionally delivered B7-H3-CAR T cells in children and young adults with primary CNS tumors or diffuse midline gliomas.	I	Central Nervous System Neoplasms|Atypical Teratoid/Rhabdoid Tumor|Diffuse Midline Glioma, H3 K27M-Mutant|Ependymoma|High Grade Glioma|Glioblastoma|Medulloblastoma	R
NCT05474378	Evaluate the manufacturing feasibility and safety of intrathecal B7-H3 CAR T cell delivery in adults with recurrent IDH-wild-type glioblastoma.	I	Brain and Nervous System	R
NCT05366179	Assess the safety of B7-H3 CAR T cells (CAR.B7-H3T) in patients with glioblastoma.	I	Glioblastoma Multiforme	R
NCT06815029	Evaluate the safety, tolerability, and optimal dose of TGFβR2KO/IL13Rα2 CAR T cells delivered intracranially in recurrent or progressive glioblastoma or IDH-mutant astrocytoma.	I	Recurrent Astrocytoma, IDH-Mutant, Grade 3|Recurrent Astrocytoma, IDH-Mutant, Grade 4|Recurrent Glioblastoma	R
NCT05660369	Determine the optimal dose and safety of CARv3-TEAM-E T cells for treating patients with glioblastoma.	I	Glioblastoma|Malignant Glioma|Recurrent Glioblastoma|Recurrent Glioma	R

## Data Availability

The data presented in this study are available upon request from the corresponding author due to privacy and ethical restrictions.

## Abbreviations

The following abbreviations are used in this manuscript:

(^18^F)FET	O-(2-[^18^F]fluoroethyl)-L-tyrosine
5-FC	5-fluorocytosine
5-FU	5-fluorouracil
APC	antigen-presenting cell
BBB	blood–brain barrier
BCNU	carmustine
BELOB	bevacizumab and lomustine in recurrent glioblastoma
BEV	bevacizumab
CAR	chimeric antigen receptor
CCNU	lomustine
CNS	central nervous system
CRCs	Chromatin-remodeling complexes
CpG	cytosine–guanine–dinucleotide
DC	dendritic cell
DD	dose-dense
*EGFR*	epidermal growth factor receptor
EOR	extent of resection
FISH	fluorescence in situ hybridization
GBM	glioblastoma
GSC	glioma stem cells
H-1PV	H-1 parvovirus
HER2	human epidermal growth factor 2
HIF-1α	hypoxia-inducible transcription-factor-1α
*IDH*	isocitrate dehydrogenase
IL-13	interleukin-13
LAT1	L-type amino acid transporter
LITT	laser interstitial thermal therapy
MGMT	O^6^-methylguanine-DNA methyltransferase
MRI	magnetic resonance imaging
ndGBM	newly diagnosed glioblastoma
NK	natural killer
OS	overall survival
PD-1	programmed death-1
PD-L1	programmed cell death ligand-1
PET	positron emission tomography
PFS	progression-free survival
PSMA	prostate-specific membrane antigen
*PTEN*	phosphatase and tensin homolog
QoL	quality of life
REGAL	REcentin™ in Glioblastoma Alone and with Lomustine
REGOMA	regorafenib glioblastoma
rGBM	recurrent glioblastoma
SOC	standard of care
TAMS	tumor-associated macrophages
*TERT*	telomerase reverse transcriptase
TME	tumor microenvironment
TMZ	temozolomide
*TP53*	tumor protein p53
TTF	tumor treating fields
reRT	reirradiation
WHO	World Health Organization
